# Clinical Radiobiology of Fast Neutron Therapy: What Was Learnt?

**DOI:** 10.3389/fonc.2020.01537

**Published:** 2020-09-15

**Authors:** Bleddyn Jones

**Affiliations:** ^1^Gray Laboratory, Department of Oncology, University of Oxford, Oxford, United Kingdom; ^2^Green Templeton College, University of Oxford, Oxford, United Kingdom; ^3^University College Department of Medical Physics & Biomedical Engineering, London, United Kingdom

**Keywords:** neutron therapy, radiobiology, radiotherapy, hadron, high LET, RBE

## Abstract

Neutron therapy was developed from neutron radiobiology experiments, and had identified a higher cell kill per unit dose and an accompanying reduction in oxygen dependency. But experts such as Hal Gray were sceptical about clinical applications, for good reasons. Gray knew that the increase in relative biological effectiveness (RBE) with dose fall-off could produce marked clinical limitations. After many years of research, this treatment did not produce the expected gains in tumour control relative to normal tissue toxicity, as predicted by Gray. More detailed reasons for this are discussed in this paper. Neutrons do not have Bragg peaks and so did not selectively spare many tissues from radiation exposure; the constant neutron RBE tumour prescription values did not represent the probable higher RBE values in late-reacting tissues with low α/β values; the inevitable increase in RBE as dose falls along a beam would also contribute to greater toxicity than in a similar megavoltage photon beam. Some tissues such as the central nervous system white matter had the highest RBEs partly because of the higher percentage hydrogen content in lipid-containing molecules. All the above factors contributed to disappointing clinical results found in a series of randomised controlled studies at many treatment centres, although at the time they were performed, neutron therapy was in a catch-up phase with photon-based treatments. Their findings are summarised along with their technical aspects and fractionation choices. Better understanding of fast neutron experiments and therapy has been gained through relatively simple mathematical models—using the biological effective dose concept and incorporating the RBEmax and RBEmin parameters (the limits of RBE at low and high dose, respectively—as shown in the Appendix). The RBE itself can then vary between these limits according to the dose per fraction used. These approaches provide useful insights into the problems that can occur in proton and ion beam therapy and how they may be optimised. This is because neutron ionisations in living tissues are mainly caused by recoil protons of energy proportional to the neutron energy: these are close to the proton energies that occur close to the Bragg peak region. To some extent, neutron RBE studies contain the highest RBE ranges found within proton and ion beams near Bragg peaks. In retrospect, neutrons were a useful radiobiological tool that has continued to inform the scientific and clinical community about the essential radiobiological principles of all forms of high linear energy transfer therapy. Neutron radiobiology and its implications should be taught on training courses and studied closely by clinicians, physicists, and biologists engaged in particle beam therapies.

## Introduction

To understand the motivation for neutron therapy and its research history, readers from different academic backgrounds require a good working knowledge of radiobiology, as well as insights into the historical development of x-ray (or photon)—based radiotherapy. This is important because fast neutron therapy was essentially competing with best practice using x-rays (or photons).

This review focuses on the clinical radiobiology aspects of fast neutron therapy, which considers the interaction between radiobiology discoveries and their applications in treatment, with radiobiological modelling used not only as analytical tool, but also as a guide to clinical practice. It is not meant to be a comprehensive review of treatment results or the quite separate topic of experimental neutron radiobiology.

It is essential to consider the progressive technical development of photon-based treatments (most of which have been beneficial), as well as the present shift to greater use of charged particle therapy and place neutron therapy within these different radiation modalities. Neutrons are hadrons and are sometimes included within the general concept of hadron therapy, which can cause confusion because neutrons have no electrostatic charge. Readers should be aware of the important aspects of dose distribution for neutrons and photons (a progressive attenuation with tissue distance or depth) but that protons and ions have Bragg peaks due not only to their charge but also a velocity reduction along particle tracks. The term *fast neutrons* refers to neutrons that have sufficient energy to cause recoil protons that ionise materials. They should be distinguished from very low energy thermal neutrons found in nuclear reactors.

### A Brief Synopsis of External Beam Radiotherapy Development

In order to compare the effectiveness of neutrons with x-rays (photons), the following information must be understood, especially the fact that more sophisticated technical developments in treatment delivery occurred sooner in the case of x-rays than with neutrons. Technical developments were mainly based on the achievement of higher photon energies, which increased the range or tissue depth, while also for megavoltage photons reducing the skin or entry dose before attaining full secondary electronic equilibrium at the maximum dose, followed by pseudoexponential attenuation with increasing tissue depth. Increasing the photon energy to the megavoltage range was also accompanied by more uniform tissue attenuation depending on the electron density rather than the actual atomic composition at lower energies. Improvements in imaging techniques further improved radiotherapy targeting, and it became possible to superimpose radiation depth dose curves in three-dimensional (3-D) space on the relevant scan or even combination of fused scan images.

During the 1950s and 1960s, external radiation beams progressed to the megavoltage energy range by use of 60-cobalt units and later increasing use of electron linear accelerators, which depended on the cavity magnetron principles originally used in radar applications (1).

With such competition, the already existing cyclotron accelerators were only rarely used for clinical neutron applications until a later time. For example, acceleration of protons onto beryllium targets to produce fast neutrons for experimental and clinical studies (2). Megavoltage photon radiation produced improved depth dose curves, enabling deeper seated tumour to be treated and with fewer beams, thus reducing the integral dose compared to when lower voltage beams were used.

Along with the computing advances mentioned previously, during the 1990s it became possible to shape each individual beam in order to match the clinically defined target by introducing variable strips of shielding metal in the accelerator collimation system. This became known as conformal radiotherapy. A UK randomised clinical trial showed that normal tissue side effects were reduced because of the large reduction in normal tissue volume irradiated to a high dose but without a reduction in prostate cancer tumour control with long-term analysis (3). The beam control possibilities improved further by using the multileaf collimator (MLC) to vary the beam intensity along its profile: this became known as intensity-modulated radiotherapy (IMRT), which further improved the degree of dose conformity to the defined target volume. Again, randomised studies of IMRT showed significant improvement of parotid gland function while treating head and neck cancer (4). However, in order to achieve the best available conformity to the target volume, the net effect was to increase the amount of tissue exposed to medium or lower doses, with some potential for causing more subtle later effects such as cancer induction or late vascular effects over subsequent time periods of 5 to 30 years. More recently, developments include robotically controlled small linacs, offering rapid changes in beam direction and intensity modulation of beamlets, which can further improve the conformity index and can be used with more precise body immobilisation techniques and state-of-the-art image guidance techniques (5). These treatments, depending on the dose distributions achieved, can be given in fewer treatments (hypofractionation) or even in a single session (often referred to as radiosurgery) (6).

Over the past two decades, and from a small initial base, there has been an expansion in cyclotron or synchrotron acceleration to deliver protons and light ions for cancer therapy in more than 100 hospitals worldwide (7, 8). These positively charged particles with their Bragg peaks whose tissue depths can be controlled by good energy selection coupled with detailed imaging, so that preferential energy deposition can occur in the selected cancer volume and its immediate surroundings.

**Figure 1:**
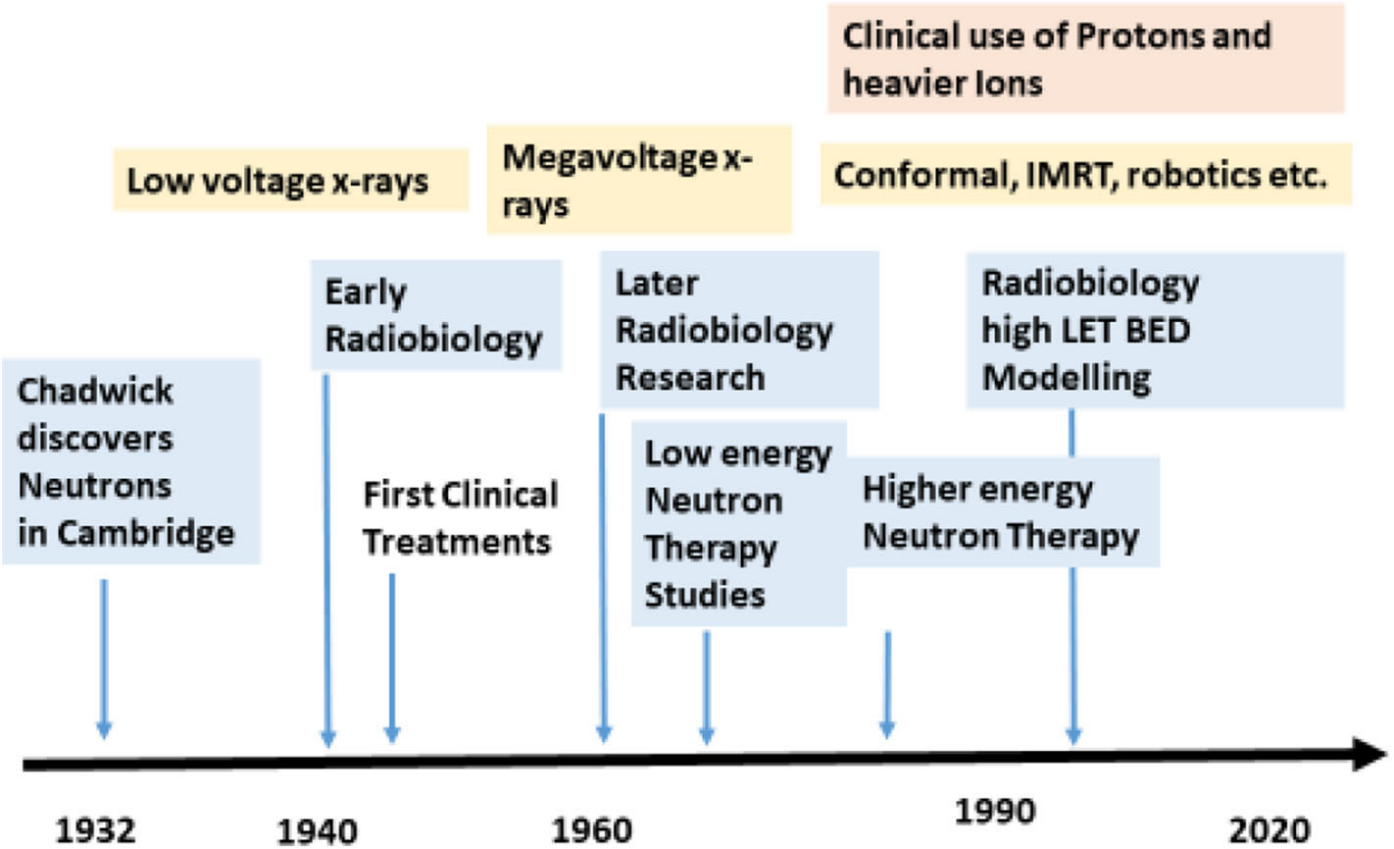
Approximate time frame for neutron research and therapy indicated by blue with x-ray/photon developments in yellow.

Figure 1 shows the relative time frames of x-rays–based and neutron therapy along with its associated radiobiology.

### Biological Effects

The above discussion has been concerned with the physical aspects of radiotherapy, but the important biological effects must be considered next. Neutrons have higher biological effects, quantified by the relative biological effectiveness (RBE) concept. RBE is the ratio of the dose of the reference radiation divided by the dose of the neutron radiation for the same biological effect. It follows that the reference radiation dose must be divided by the RBE in order to provide the equivalent (and lower) neutron dose, as shown:

(1) $$\begin{array}{r} \text{RBE}=\frac{\text{Dose }\left(\text{photons}\right)}{\text{Dose }\left(\text{neutrons}\right)} \end{array}$$

(2) $$\begin{array}{r} \text{Dose}\left(\text{neutrons}\right)=\frac{\text{Dose }\left(\text{photons}\right)}{\text{RBE}} \end{array}$$

Research studies showed that the biological effects of photon radiation varied not only with dose but also the dose rate, the degree of dose fractionation (in which the dose can be split in time into different treatment sessions), the chemical environment of the cells (some chemicals protecting and others sensitising radiation by influencing the yield of free radicals), and also the “quality” of the radiation. The latter refers to the linear energy transfer (LET) characteristics of a radiation, which depends not only on the nature of the radiation (e.g., photon or hadron), its energy (lower energies confer higher LET), and the total nuclear electrostatic charge given by the *Z* number of each element. Photons ionise by means of electronic interactions, forming secondary electrons in matter. Lower energy electrons converted by 10 to 100 keV photons have higher LET and biological effects than those in the megavoltage range (9).

### Physical Interactions of Neutrons With Matter

**Figure 2:**
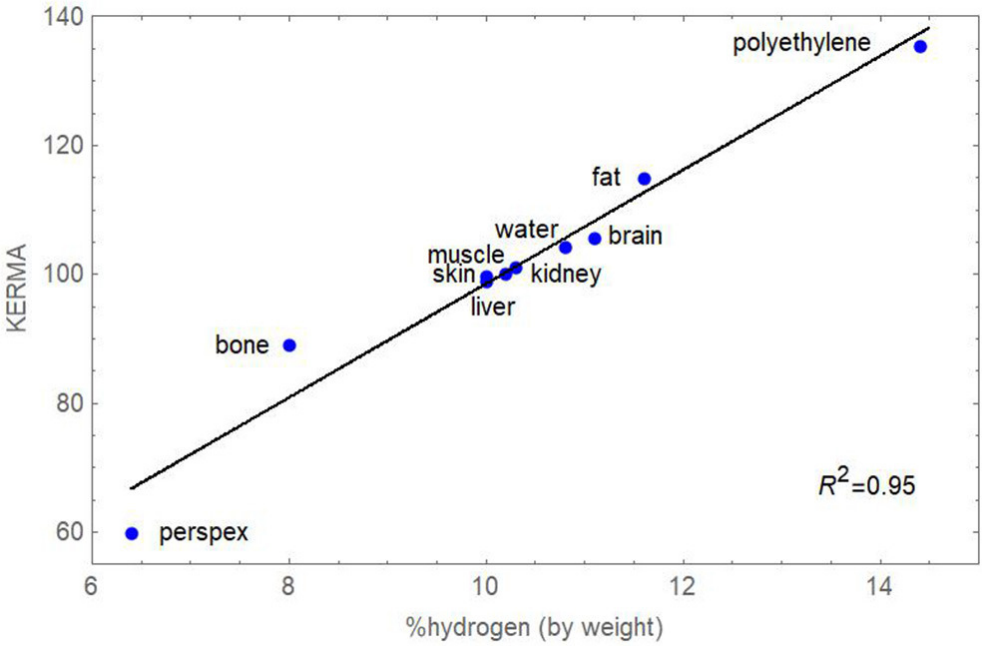
Plot of percentage hydrogen (by weight) and KERMA for a range of materials and tissues exposed to five different fast neutron beams. Standard errors are not shown for individual points since they are small (average 0.78, the largest being 2.1 for bone). Data obtained from Awschalom, Rosenberg & Mravca (10).

Neutrons do not interact with atomic electrons like photons do, but instead the uncharged fast neutrons efficiently interact with hydrogen nuclei, producing recoil protons that ionise. As well as water, other biological macromolecules containing a relatively high proportion of water hydrogen include lipids and lipoproteins, so that neutrons will deposit more energy in tissues that contain, for example myelin and sphingomyelin in brain and spinal cord white matter, and of course in body fat. Fatty tissue contains long fatty acid chains CH_3_-(CH_2_)_N_-COOH, and exists in the connective tissues that surround many organs of the body and through which the vital blood vessels pass. The abdomen contains a large intraperitoneal fat pad called the omentum, which is in intimate contact with the bowel. The KERMA (kinetic energy released per unit mass) in lipid-containing tissues can exceed that in water, which contains a relatively high proportion of hydrogen (11.1% by weight), resulting in increased local dose and bioeffectiveness. Previously published data from a detailed study (10) were combined to show a linear relationship between the percentage of hydrogen (%H) contained in some materials and tissues and KERMA. The increase in KERMA is ~8.8% per unit increase in the %H as shown in Figure 2.

## Neutron Therapy and Radiobiology

Some general comments are appropriate at this stage in order to fully appreciate the issues discussed below. Experts tend to be dismissive of the history of fast neutron therapy, because of the disappointing clinical outcomes, and many clinical specialists were afterwards sceptical about using any form of particle therapy emerging from a cyclotron. To understand this topic fully, the radiobiology and therapy have to be taken together, with a later description of advances in the radiobiological understanding, which occurred that followed with time.

### Why Study Neutron Therapy and Radiobiology?

It is now vital that lessons learnt from fast neutrons' radiobiology experiments and from the clinical trials are acknowledged and that these invaluable data should be part of the essential background required for making progress with other hadrons (protons and ion beam therapy). Ignoring the facts that have emerged may lead to a repetition of disappointing clinical results and eventual reduction of referrals for proton and ion beam therapy. It is vital to understand that most of neutron ionisation occurs from recoil protons, as well as some nuclear fragments, so their radiobiological features, including their RBE values, will be similar to those of a proton beam in the Bragg peak region where the LET and dose increase substantially. This fact has not been sufficiently well-recognised within the proton therapy community until very recently (11, 12), and RBE values in the fast neutron range have been found at the end of spread-out Bragg peaks in the human lung (13). These aspects should be borne in mind when reading the remainder of this review, where it will become apparent that RBE issues cannot be dismissed lightly in any form of particle therapy.

### The Historical Case for Neutron Therapy

The medical rationale for fast neutron therapy of cancer was based on initial scientific *in vitro* experimental evidence of more efficient cell killing per unit dose and a reduced dependency on tissue oxygen tension. The history of neutron therapy illustrates the problems that can arise when partial scientific knowledge is used in an attempt to improve the treatment of a complex biological condition such as cancer; it is self-evident that the sterilisation of cancer cells by a radiation technique in an *in vitro* laboratory experiment is more likely to be successful than the elimination of a malignant tumour situated close to essential organs/tissues of the body. For future radiotherapy developments, particularly the use of proton and ion beam therapy, this history is important.

**Figure 3:**
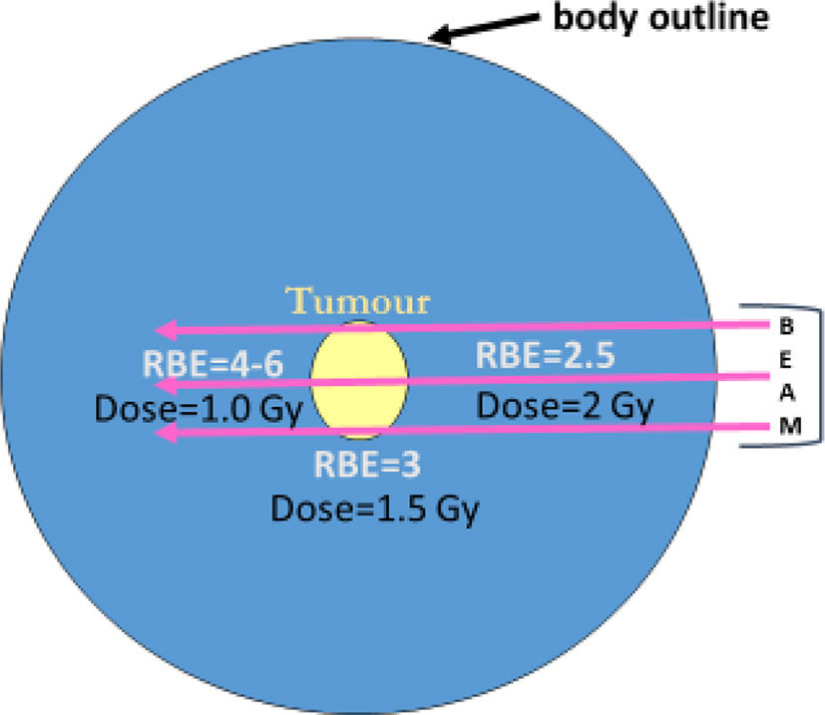
Schematic diagram of external neutron beams passing through a tumour (yellow) with progressive increase in RBE and dose reduction. Prescription of radiation used RBE of 3 at the tumour depth and assumes this to be operative at all other points within a patient, but the RBE is increasing where dose fall-off occurs beyond the tumour target as illustrated.

In 1940, Gray et al. (14) had shown that it was possible to achieve the same level of biological effect with a lower dose of neutrons than with γ- or x-rays. This difference was quantified by the RBE. Fast neutron RBE values of 1.5 to 5 were found in a variety of biological systems (bacteria, plants, transplanted animal cancers). The immediate inference was that neutrons would be ideal for cancer therapy, yet the first human treatments in the United States (again in the early 1940s) showed marked toxicity, because the relationship between the exposure dose and RBE had not yet been identified (15). Gray remained sceptical and realised that neutron therapy may not be successful because of the spatial changes in RBE, which would inevitably occur within the human body. He knew that RBE was inversely related to dose, so that dose fall-off with distance along a neutron beam would inevitably be accompanied by higher RBE values in normal tissues beyond any cancer target, as shown in Figure 3. His opinion on neutrons had devastating personal consequences for radiobiology and radiotherapy, because he was dismissed from his post as director of radiotherapy physics at the Hammersmith Hospital, although his career was rescued by the philanthropic formation of the Gray laboratory elsewhere in London (16) and is also now remembered by the SI unit of absorbed dose in units of Grays. Gray believed that neutrons were an important tool for research, for the investigation of high LET effects, but should not necessarily be used for treatment. He was eventually proved correct, and the wealth of fast neutron experimental data (much of it performed in the United Kingdom) probably provides the best insights into high LET phenomena, especially that the inverse dose per fraction effect on RBE is especially marked in late-reacting normal tissues when compared with acute-reacting tissues.

### Further Experimental Work

Further interest arose because of the discovery that high LET radiations, e.g., fast neutrons, with increased clustering of ionisation events along micrometre distances of their tracks, are less dependent than are x-rays on the presence of oxygen to produce cell death (oxygen essentially amplifies low-LET ionisations by increasing the yield of reactive free radicals in solution). The previous work of Gray and others had shown that many cancers contained zones of very low oxygen tension, which were considered an important cause of radioresistance. To overcome this problem, high-pressure oxygen (HPO) was used in experimental radiotherapy from the 1960s for several decades. In the United Kingdom, the initial results in animal experimental systems were impressive (17). In clinical practice, HPO had many disadvantages, because patients had to be placed within HPO tanks or chambers, and there was no overall improvement in patient survival, although some tumour types were better controlled (18). An attractive alternative to HPO was the use cyclotrons to accelerate protons—to around 16 to 20 MeV to produce fast neutrons with high LET properties and reduced oxygen dependency for cell killing within cancers. It was argued by the neutron enthusiasts that the use of HPO chambers would be unnecessary if neutron therapy would be used more extensively.

### Clinical Research

The research efforts were complicated by there being several generations of technical equipment initially based on static beams, followed later with rotating gantries (19) and finally higher neutron energies in order to match the treatment geometry and depth dose characteristics of clinical megavoltage treatments. This technical evolution occurred at much later times than with photons, so neutron therapy was continually in a catch-up state.

Many developed countries started neutron therapy research programs, often based in single institutions and concentrating on treating rare tumours such as sarcomas. Some encountered severe tissue complications and were discontinued. The usefulness of neutron therapy could not be gauged from the emerging data sets, containing small numbers of patients in each tumour class, so it eventually became necessary to perform randomised studies on more commonly occurring cancers in order to determine the role of fast neutrons in the treatment of cancer.

### The UK Medical Research Council Trials

The UK Medical Research Council (with other charitable contributions) funded three important sequential projects to investigate the usefulness of fast neutron therapy (20), from the late 1960s onwards until around 1992. Further work on the radiobiology also continued in parallel.

1. At Hammersmith (University of London), clinical studies were conducted by Dr. Mary Catterall with initial promise using a geometrically limited fixed horizontal beam (21, 22). Despite clear evidence that the neutron RBE was inversely related to dose per fraction in a wide variety of animal tissues, the clinical dose prescriptions used a fixed RBE. There was no 3-D dose computing availability for a more sophisticated approach. Accordingly, the dose plan took no account of the increase in RBE in normal tissues beyond the tumour, where lower doses would inevitably increase the RBE. The treatment schedules used 1.5 Gy three times per week. Promising results were reported for parotid gland and air sinus tumours, and the incidence of complications appeared to be reducing with better dose selection. A randomised trial comparing outcomes with conventional photon-based treatments involved control patients treated with x-rays or cobalt beams at other hospitals without defined protocols so that a wide range of doses, including some that were unsuitable, were used. From that time on, cancer trials were conducted with greater rigor.
2. Arnott and Duncan at Edinburgh Royal Infirmary (University of Edinburgh) used stricter “in-house” randomised trials to compare megavoltage x-rays (with their superior tissue penetration) with relatively poorly penetrating fast neutrons (as in Hammersmith), but for both radiation classes, the beams could be rotated on a gantry. The photon-treated controls were consequently treated with fewer beams per treatment plan, and the neutron treatments contained up to seven fields per plan, thus increasing the integral dose of neutrons. Treatments were supervised by site-specialised cancer experts with good academic supervision. Although in some instances improved local cancer control was achieved, enhanced normal tissue toxicity was also reported. It is interesting to note that the neutron dose per fraction of 0.9 Gy, given five times per week, was lower than that used in Hammersmith, thus inevitably increasing the RBE and its normal tissue consequences.
3. Errington and Warenius at Clatterbridge (University of Liverpool) used an extended fast neutron energy (obtained using 64-MeV protons from a cyclotron) with depth doses equivalent to 5-MeV x-rays in randomised trials, some of which were jointly undertaken with Seattle and Fermilab (USA). The dose per fraction chosen was the same as Hammersmith and treatment delivered 3 days per week to relatively advanced T3 head and neck and pelvic cancers. Compared with the photon treatments, neutron therapy toxicity was not increased, but there was no improvement in tumour control, and rather surprisingly, the metastatic rate appeared to be increased (perhaps due to the intermittent dose fractionation used).

Taken together, all these trials showed that neutrons conferred no clinical advantage, as comprehensively summarised by Duncan (20), which should be consulted for further details and references.

### Other Neutron Therapy Studies

In many other countries, relatively low energy neutrons had been tried without recourse to formal trials and with little convincing success, although a small randomised trial reported by Laramore et al. (23) showed benefits for neutrons in the control of unresectable cancers of the parotid gland, which was similar to the findings at Hammersmith. In this trial, it is possible that a higher dose of x-rays with an electron boost in the control arm might have produced the same result. The actual tumour RBE, which was probably higher than that used in the prescription since the adenoid cystic carcinomas used in the trial, was very slow growing, but some tumours may contain a high fatty acid concentration, which could increase neutron KERMA (24). Relatively superficial air sinus cancers were also thought to be better controlled by neutron therapy, although there was always concern that neutrons were particularly damaging to the underlying brain tissues, where it had been identified previously at Hammersmith that the white matter RBE was around 5 rather than 3, again for reasons already identified above.

Some later neutron therapy research used polyethylene and iron MLCs, which initially started in Seattle (25), and then in Essen and Detroit, where intensity-modulated neutron therapy was introduced (using the MLC), especially for the treatment of prostate cancer (26). However, the inevitable increase in low-dose volume (often referred to in some countries as the low-dose “bath”) does raise concerns about integral dose concerns and especially since the Edinburgh group had by 2006 reported a significant increase in radiation-induced sarcomas over a period of 30 years (27), although such a risk could perhaps be discounted in elderly patients. From that time onwards, there has been increasing competition for prostate cancer treatments using brachytherapy, proton therapy, and robotic surgery.

## Retrospective Insights

In retrospect, neutron therapy failed to match its original promise for the following physical and radiobiological reasons:

- Routine absorbed dose computations did not include the highly efficient neutron interaction with hydrogen, resulting in higher energy release in hydrogen rich tissues such as brain white matter and fat, which surrounds most important organs and is closely associated with their blood supply.
- Dismissal of the well-established finding of RBE variations in different tissues and its important increase with a falling dose, which mitigates the effect of a reduction in physical dose beyond a cancer.
- The appreciation that RBE also varies with cell proliferation rate, so that slow-growing tumours have higher values. It is the slow-growing stem cells and vascular cells that contribute to the severe normal tissue damage at extended time periods after irradiation.

### Some Generic Knowledge

Several generic improvements emerged as a consequence what was found useful in the neutron therapy trials. Nearly all clinical trials in radiotherapy after that time followed the Clatterbridge UK and North American RTOG neutron trials by inclusion of data monitoring committees, as well as rigorous quality assurance (QA) systems for dosimetry, as well as clinical standards. When the detailed QA systems established for neutrons were applied as a result of discussion within the PTCOG (Particle Therapy Co-operative Group), the study coordinated at Loma Linda showed potentially significant discrepancies in some major proton centres and which required correction by up to 7% (28). Furthermore, when the UK Hospital Physicists decided to compare dosimetry standards at all UK radiotherapy centres by using thermoluminescent dosimetry in the same body phantom (29), the Clatterbridge Hospital results were closest to the dosimeter used in the UK National Physical Laboratory. This was a good example of how the discipline of the neutron trials exerted an influence on the conduct of radiotherapy physics in one hospital.

### Mathematical Modelling

The early neutron radiobiology was well summarised by Bewley (30) and remains relevant to particle therapy. In 1998, mathematical modelling, which included RBE within biological effective dose (BED) equations, showed that neutrons would only be capable of improving clinical outcomes in the case of the treatment geometry provided by very superficial cancers with little normal tissue coverage (31), exactly the condition for air sinus and parotid tumours. The further extension of BED equations that contain RBE allowances in many radiobiological settings has the potential for guiding clinical practice (32), and the relevant equations are given below and in the Appendix. These BED equations used the RBEmax and RBEmin concepts in conjunction with the reference radiation tissue α/β ratio, which are known with greater confidence than the much higher neutron α/β values.

The standard low LET BED equation is

(3) $$\begin{array}{r} {\text{BED}=\text{D}}_{\text{L}}\left(1+\frac{d_{L}}{{\left(\frac{\alpha }{\beta }\right)}_{L}}\right), \end{array}$$

where *D* symbols refer to total doses and *d* the dose per fraction given in *n* treatments (or fractions) with subscripts L and H used to denote high and low LET radiations, respectively, the latter being the reference radiation for RBE estimations.

For high LET radiations, Equation 10 can be modified (31, 33) by inclusion of RBEmax and RBEmin (formal definitions are given in the Appendix) where to be

(4) $$\begin{array}{r} \text{BED}=D_{H}\left(\text{RBEmax}+\frac{{\text{RBEmin}}^{2}d_{H}}{{\left(\frac{\alpha }{\beta }\right)}_{L}}\right) \end{array}$$

Isoeffect calculations then use the equality:

(5) $$\begin{array}{r} D_{L}\left(1+\frac{d_{L}}{{\left(\frac{\alpha }{\beta }\right)}_{L}}\right)=D_{H}\left(\text{RBEmax}+\frac{{\text{RBEmin}}^{2}d_{H}}{{\left(\frac{\alpha }{\beta }\right)}_{L}}\right) \end{array}$$

By definition, *D*_L_ = n_L_.d_L_, and *D*_H_ = n_H_.d_H_, so that any two schedules with the same number of fractions can be either compared or equated for an isoeffect as in Equation 5.

For single doses, Equation 5 is modified to be:

(6) $$\begin{array}{r} d_{L}\left(1+\frac{d_{L}}{{\left(\frac{\alpha }{\beta }\right)}_{L}}\right)=d_{H}\left(\text{RBEmax}+\frac{{\text{RBEmin}}^{2}d_{H}}{{\left(\frac{\alpha }{\beta }\right)}_{L}}\;\right) \end{array}$$

Further details on obtaining an RBE at any dose per fraction are given in the Appendix.

### Important Radiobiological Concepts That Emerged From Fast Neutron Experimental Studies

**Figure 4:**
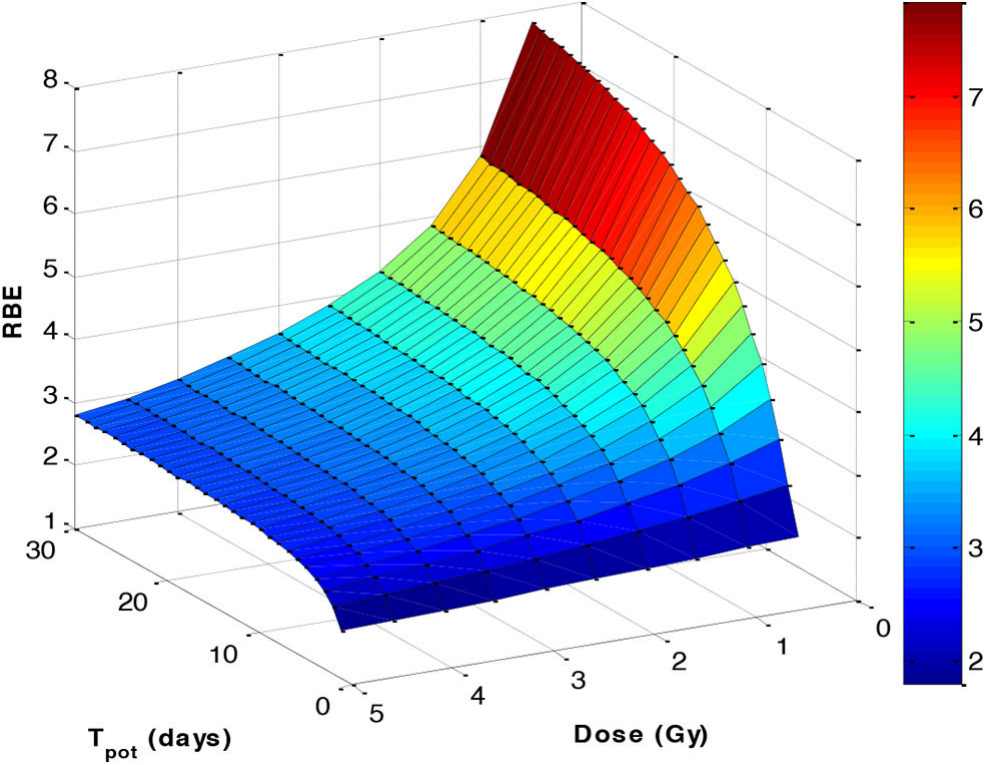
A 3-D plot based on the Batterman data set (35), assuming a 90% cell loss factor to convert the volume doubling time to an estimated potential doubling time (*T*_pot_), which is itself inversely related to the α/β ratio by a function α/β = 48.9/*T*_pot_. The RBE was then estimated using the linear-quadratic formulations given in the Appendix.

Radiobiology studies had already shown high neutron RBE values, which varied inversely with dose, reductions in oxygen enhancement ratio (OER), and cell cycle phase dependency, and with greater dose per fraction insensitivity, with similar findings for heavier ions (34). Also, Batterman et al. (35) had shown that human tumour RBE values were related to their volumetric doubling times and which can be related to tumour potential doubling times (Figure 4) (36). Extended analysis of the fast neutron experiments at Hammersmith and Clatterbridge continued in more recent times, providing many informative reports and interpreted using the linear-quadratic model of radiation effect as in Carabe-Fernandez et al. (33, 37) and Jones et al. (38), which model the well-recognised inverse dose per fraction and RBE effects (using the RBEmax and RBEmin concepts), in different tissues represented by their characteristic α/β ratios. Acute-reacting tissues, such as oesophageal mucosal reactions, show almost no change in RBE with dose per fraction, but later-reacting tissue effects in skin, lung, and kidney show greater changes in RBE with dose per fraction.

**Figure 5:**
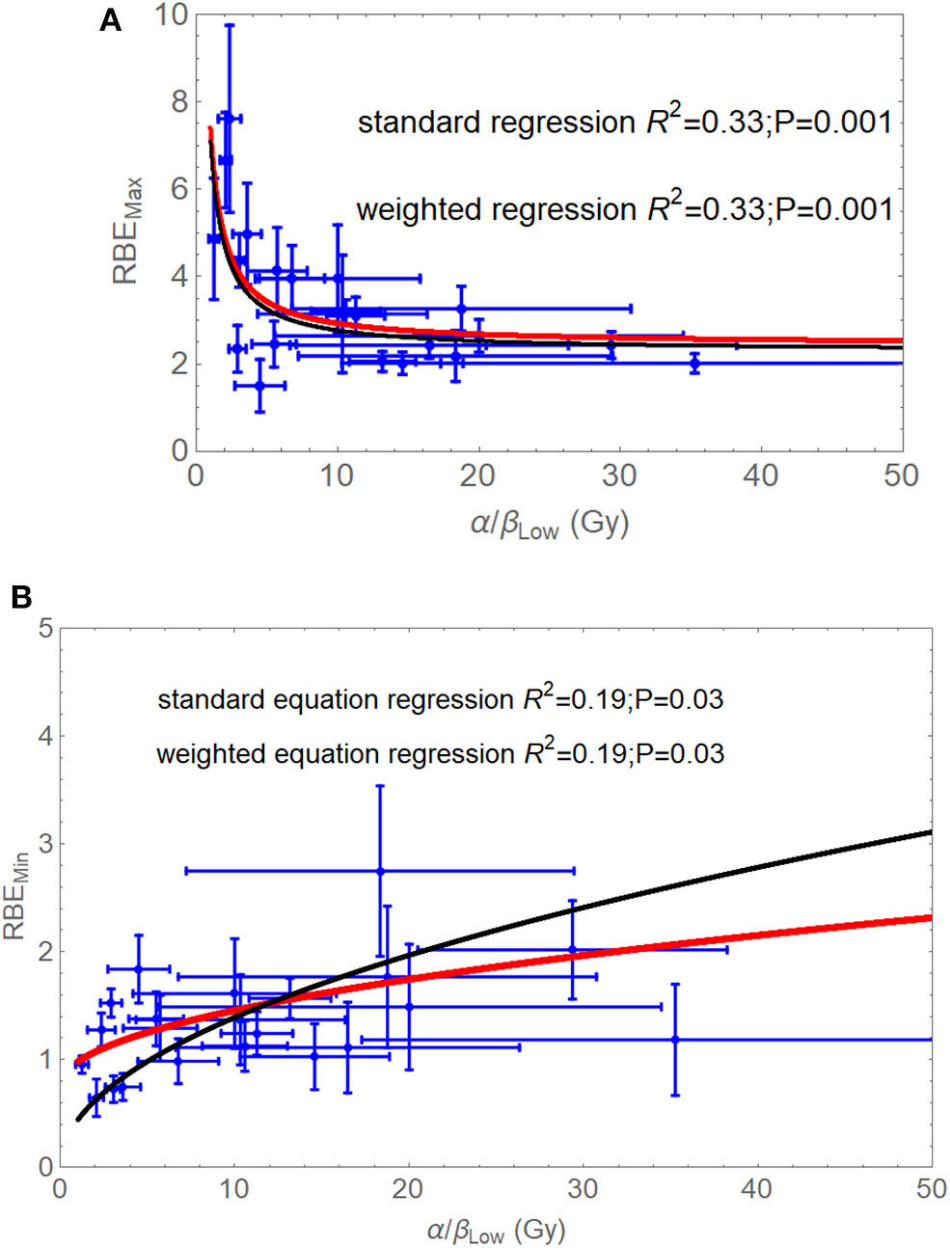
**(A,B)** Graphical displays of RBE_max_ **(A)** and RBE_min_ **(B)** plotted with respect to the reference low LET α/β ratio, as adapted from Jones et al. (38). Least squares fitting are **(A)** RBEmax = 2.43 + 4.97/(α/β)_L_ (no standard error weighting) and RBEmax = 2.29 + 4.81/(α/β)_L_ (with standard error weighting), **(B)** RBEmin = 0.76 + 0.22√(α/β)_L_ (no standard error weighting), and RBEmin = 0.73 + 0.19√(α/β)_L_ (with standard error weighting). Modified from Jones et al. (38).

**Figure 6:**
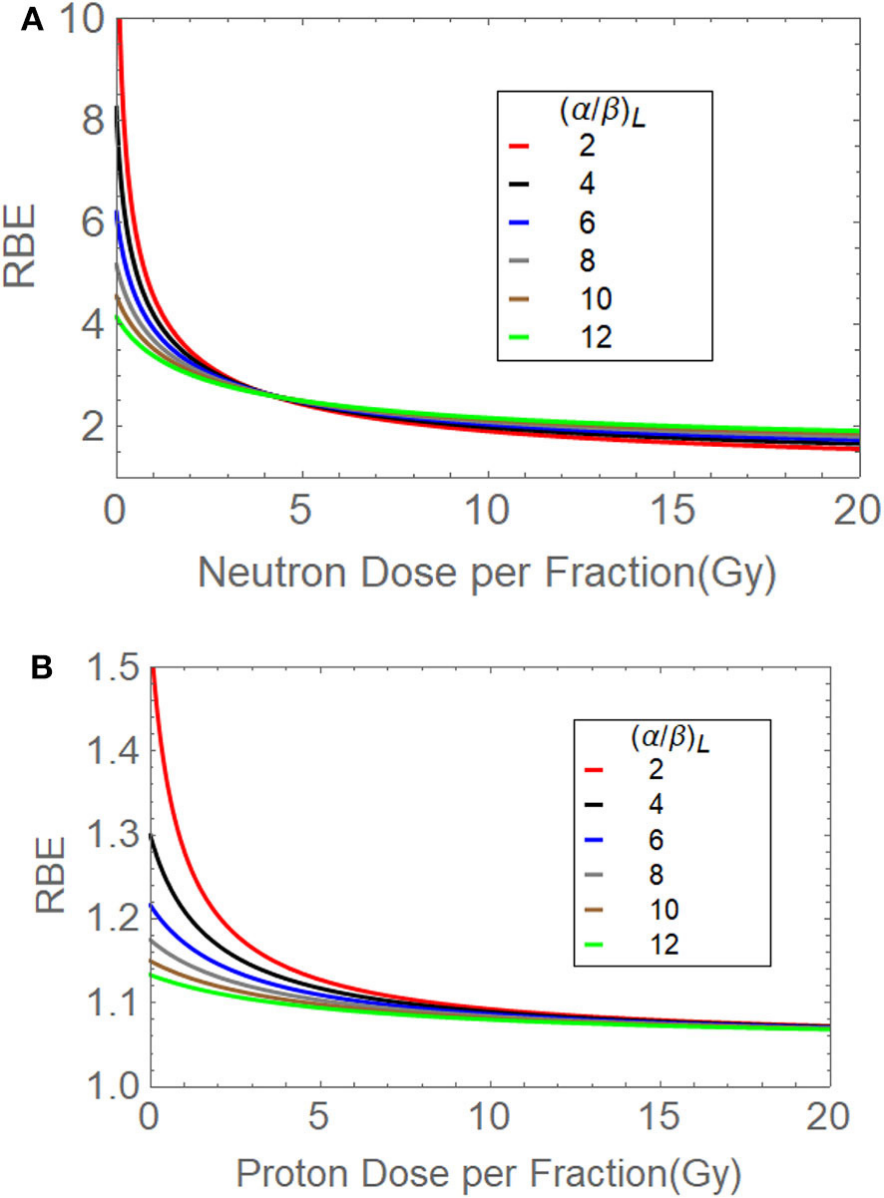
**(A,B)** The relationships between **(A)** fast neutron dose per fraction and RBE for different α/β ratios, and **(B)** the transformation of the above to provide a near-flat response for α/β of 10 and 12 Gy to simulate proton data by modification of RBEmax and RBEmin. The red curves suggest how brain and spinal cord tissues may behave (α/β = 2 Gy), followed by a gradual change in α/β to faster-growing systems such as many rapidly growing tumour types and acute-reacting normal tissues (α/β = 10 Gy). Modified from Jones et al. (38).

The graphical fits in these articles are informative, as linear-quadratic model theory does predict (Appendix) that the RBEmax is inversely related to the α/β ratio (Figure 5A), whereas RBEmin is directly proportional to the square root of α/β (Figure 5B). By using these relationships, for the most critical low α/β (late-reacting) tissues, the RBE at low dose is highest, but changes to be the lowest RBE at high dose when compared with more rapid proliferating tissues (or tumours) with high α/β ratios that have a “flatter” response, as shown in Figure 6A. In these figures, it is important to note that the RBEmax represents the RBE at near-zero dose, forming the intercept of the curves where dose is zero, and that RBEmin is the limiting value at high dose and that always exceeds unity. If RBEmin is not used, the RBE will approach 1 at high dose, whereas in experimental normal tissue systems, RBE asymptotically approaches values of between 1.2 and 1.4 in most instances. However, some data sets do contain a limited number of RBEmin values around 1, which implies little or no increment in β and possible other influences associated with experimental design, biological variation, and the smaller influence of the reference irradiator being low KeV x-rays. RBEmin *in vitro* data can only be obtained from direct estimation of β values; the dose range cannot be as high as *in vivo* studies because of limited surviving colonies in the former.

Figure 6B used lower RBEmax and RBEmin values, iteratively adjusted to match the conventionally used proton RBE of 1.1, for α/β = 10 Gy [the α/β value of the *in vivo* jejunal crypt assay, which was mostly used to achieve this RBE value in mid-spread out Bragg peak (SOBP) (11)]. By then varying the α/β ratio to represent different tissues/tumours, the dose per fraction effect can be further estimated, although in this case there is no crossing-over of the curves. At 2 Gy per fraction, the curve for α/β = 2 Gy (normally used of central nervous tissue) provides an RBE estimated of around 1.2, which is very close to that predicted by a different proton model (39).

If the overall RBE is controlled by RBEmax and RBEmin and that these parameters are, respectively, inversely and directly related to the reference radiation α/β ratio (details given in Figure 5 legends and Appendix), then a practical clinical RBE cannot be assumed to be only related to RBEmax. Mathematical models that predict RBE from changes in only the α parameter or only from α/β are probably incomplete, because it is necessary to include specific increases in the β parameter with LET, although these are less marked than for the α parameter. Comparisons of models that predict proton RBE values have been made by Paganetti et al. (12) and Warenius et al. (40).

At Clatterbridge Hospital, Warenius and Britten (41) had identified that the respective rank orders of radiosensitivity values for photons and neutrons were different. This is a consequence of the transition of RBE between the limits provided by RBE max and RBEmin (as shown in Figure 6A) and other nonlinear effects such as the progressive reduction of RBE increments with increasing intrinsic (photon) radiosensitivity found in cellular experiments (39).

**Figure 7:**
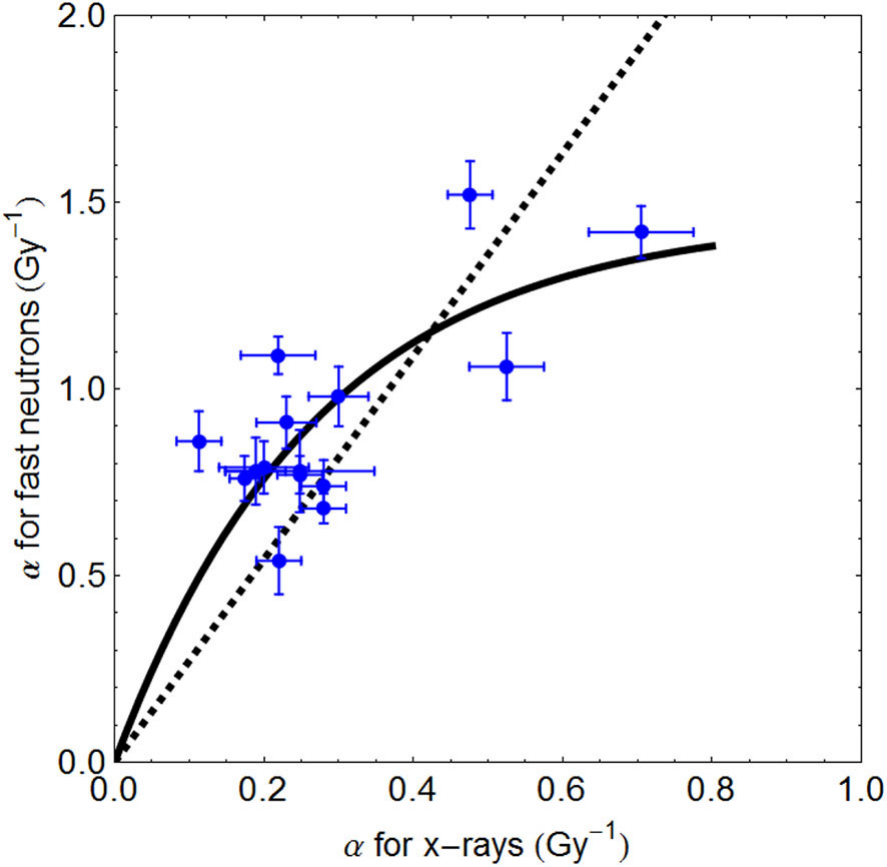
Plots of α radiosensitivity with standard errors for 5-MeV x-rays and 64-MeV neutrons. The fitted curve follow the relationship α(neutron) = 5.37/3.68(1–e^3.68α(−*x*−*ray*)^). The hatched line represents a linear fit, which would inappropriately lead to much higher α_H_ values.

It has also been shown that the LET values produced by the Clatterbridge fast neutrons increases the α-radiosensitivity parameter by a factor of 3.17, but the β parameter increases by 1.59√β on average (42). The increment in α for the Clatterbridge fast neutron and x-ray comparisons in cell lines are shown in Figure 7, where a saturation effect appears to limit the increase in radiosensitivity (39).

**Figure 8:**
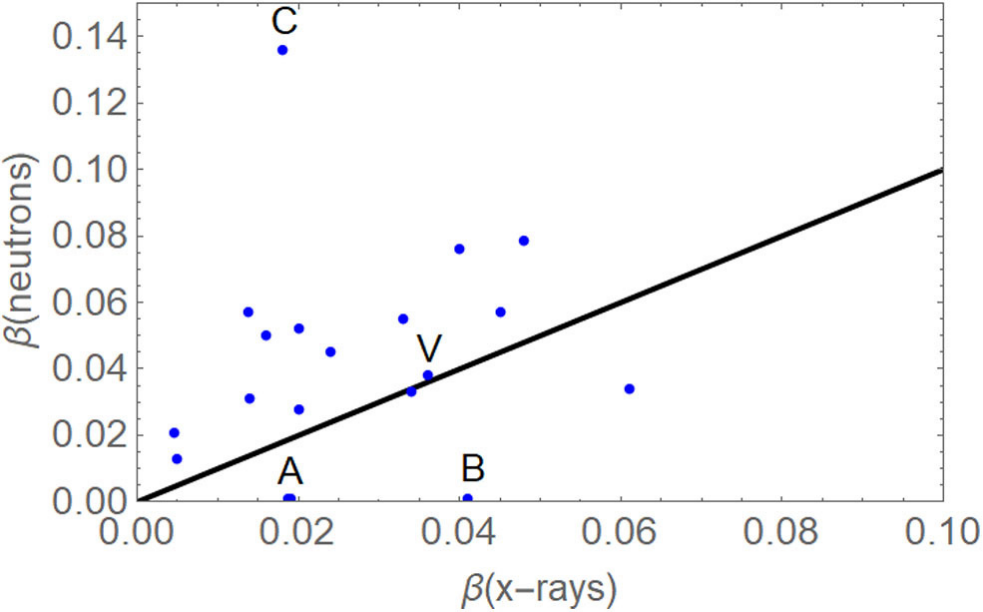
Plots of the β parameter for megavoltage photons and neutrons. Outlying cell lines with β neutrons close to zero (labelled A and B) were excluded because of probable experimental error, and the extremely high value (C) was due to known radiation repair deficiency and was also excluded. The black line will be followed if there is no change in β with increasing LET, but most nonexcluded data points are above that line. The V-79 data point (labelled V) falls just above it. Application of the sign test indicates that the hypothesis that β is the same for neutrons and megavoltage x-rays can be rejected (*p* < 0.01). Adapted from Jones (42), with no error bars for ease of viewing.

**Figure 9:**
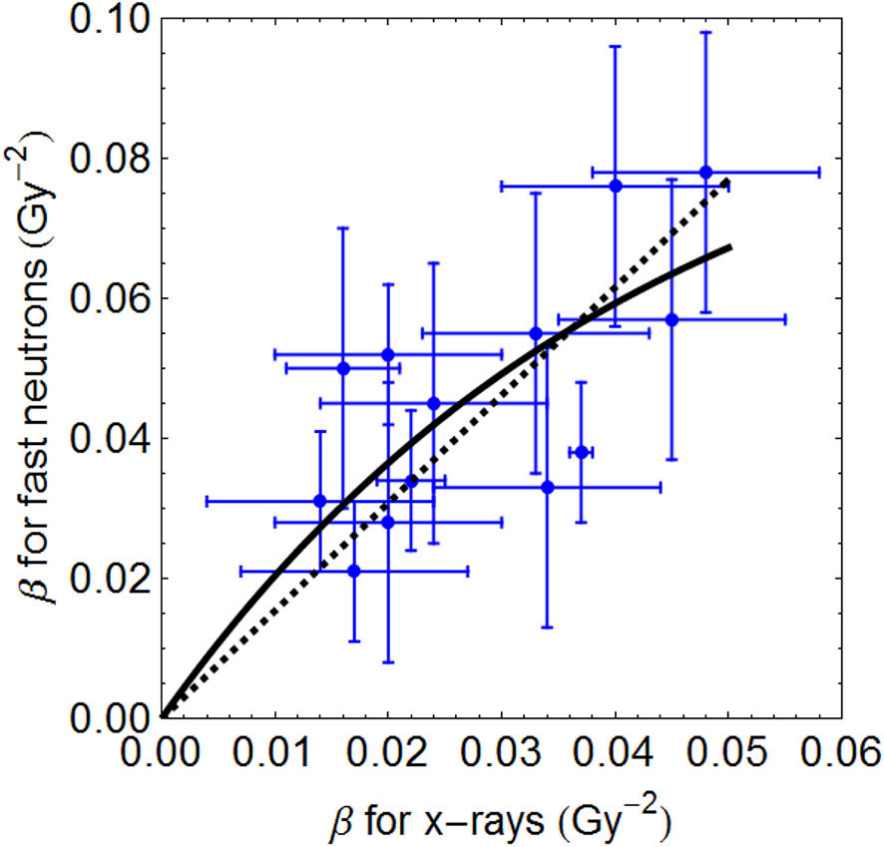
The representative data in Figure 8, with error bars and fitted with a linear function and by a saturation curve described by β (neutron) = 2.29/23.57 (1–e^−23.57β(*x*−*ray*)^). The later fit is preferable because the linear equation will continue to increase beyond acceptable limits.

Previously, it was widely thought that β did not increase significantly with LET, as evidenced in meticulous experiments involving only the V-79 cell line by Jones (43), but the Clatterbridge *in vitro* experimental neutron and x-ray data set contained over 20 cell lines, and the increase in β can be seen in Figures 8, 9 (39, 42, 44), where the V-79 line is shown as being just above the line that represents no change in the β parameter. Many proton RBE models have used the V-79 cell line without any allowance for an increment in RBE with LET and so could underestimate or even exclude use of RBEmin (12, 39, 40).

Improved BED assessments of not only neutrons but also for other ion beams have included such saturation effects (39, 44).

## Boron Neutron Capture Therapy

At present, the only promising development for neutrons is arguably boron neutron capture therapy (BNCT), a complex binary therapy that involves a low-dose exposure to thermal or epithermal neutrons, which are selectively captured by boron-labelled amino acids that avidly enter rapidly growing tumour cells. The reaction creates more intense localised ionisation by generating an α particle and a lithium ion, with tissue ranges of only around 10 μm (about one-cell diameter). The RBE issues within BNCT are further complicated by the dose being dependent on the concentration of the boron-containing compound, which is reflected in a compound biological effectiveness or CBE (45). Some pilot studies have shown promise in highly malignant brain tumours or for recurrent tumours, although in uncontrolled, highly selected patients (46, 47), and the future prospects remain uncertain, although hospital-based accelerators for thermal neutron sources could reduce the inconvenient dependency on a nuclear reactor as a source (48). Further discussion is beyond the scope of this article.

## Discussion

### The Neutron Aftermath

Following the fast neutron clinical trials, the UK funding authorities decided not to invest in further ambitious radiation projects; and as a result a general decline in UK radiotherapy and radiobiology research followed, although the Clatterbridge cyclotron was successfully adapted for ocular proton therapy. There emerged a clinical scepticism regarding the use of cyclotrons in radiotherapy and high LET radiations in general. In other countries, more progress was achieved using charged particle therapy (protons or light ions) produced from cyclotrons or the larger synchrotrons. Some of these countries had no prior experience of high-LET radiations in the form of fast neutrons.

In Japan, the government selected proton and carbon ion therapy as a patient-friendly alternative to fast neutrons, but interestingly applied neutron experimental data to link the LET and RBE for treatment prescriptions (49), although not used with a dose-per-fraction effect, and there was effectively no exit dose where further RBE increments could make a significant difference. This approach has been replaced by modifications of the MKM (microdosimetric kinetic model), which originally did not allow for the increments in β with LET (resulting in a high dose RBEmin of 1), but now β effects have been introduced as a consequence of the neutron experience (50). The crossover effects observed in Figures 4A,B appear to be consistent with clinical results of reduced side effects with increasing hypofractionation in Japan (51).

A major radiotherapy research question over the next few decades will be whether carbon ions are superior to protons in specific cancers, because of better beam ballistics and dose localisation and/or the apparent greater reduction in the oxygen effect. Suit et al. (52) have pointed out that because even the neutron studies did not show a convincing overall local tumour control improvement, it should not be expected that carbon ions will provide improved efficacy because the oxygen effect dependency is greater for spread-out carbon ion peaks than for fast neutrons (OER ratios of around 1.8–2 compared with 1.6–1.8, respectively).

The process of reoxygenation in tumours during radiotherapy for schedules lasting several weeks may be the reason why hypoxic radioresistance is not especially important with photons and may explain why the control x-ray treatments, as in the Clatterbridge studies, produced results that were at least as good as with neutrons (20). However, the identification of tumours with slow reoxygenation kinetics may yet be important because use of high LET therapy and dose escalation may then be important.

### Improvement of Neutron Bioeffect Prediction

Because fast neutron energies are part of a spectrum, in principle it is possible to predict most of the RBE by considering the recoil proton energies and then applying proton RBE models (12, 39, 40) or more complex predictive models (4, 53). These models vary in terms of the inclusion of biological data and the increase in β with LET, but most appear to give realistic values compared with the incorrect assumption that all protons have an RBE of 1.1, which is an oversimplification even at mid–spread-out Bragg peak (11). Knowledge of neutron RBE continues to be required in medicine and radiation protection and for space travel.

### Mixed Neutron and Photon Fields

Research at the Gray Laboratory led by McNally had shown nonlinear effects on cell survival when photon and neutron irradiations were used sequentially, with a short temporal separation, which would not change DNA repair significantly (54). Lower doses of neutrons dominated the effectiveness, but this influence was dependent on the level of surviving fraction. Although Zaider and Rossi (55) proposed a quadrature addition of the β terms, there does not appear to be a satisfactory method for predicting mixed beam effects. The present writer is of the opinion that because the α radiosensitivity will depend on LET in a biphasic way by following the general relationship between LET and RBE, it is suggested that LET-dependent RBE models should be used in such situations, coupled with the neutron LET spectrum. This is a complex problem. German scientists are now exploring mixed field irradiation by use of their local effect model (56). Although neutron beams normally contain γ-rays, measured experimental RBEs will include such mixed field effects.

### Neutron Carcinogenesis

The problem of neutron-induced cancers has already been mentioned (27). Further radiobiological details have been reviewed by Trott (57), because neutrons and γ-rays are released from proton and other ion beam interactions in the human body and so can increase with depth along a beam. It is also the case that the RBEmax and RBEmin concepts can be applied to radiation carcinogenesis caused by high LET radiations such as neutrons (58).

### Educational Aspects

The history of neutron experimental and clinical studies should be taught to doctors and physicists during their radiotherapy training. This is because the general principles learnt are educationally informing. These topics should be studied in greater detail by those engaged in particle beam therapies. At present, there is a curious lack of emphasis on RBE issues in proton therapy teaching courses, but regulatory bodies should insist on a good background of radiobiological understanding in high LET radiations in order to guide future clinical decision makers. Neutron experimental data sets were not examples of “wasted research,” because they have been further analysed 20 to 40 years after their original publication and have some impact on informing radiation modellers of how to improve proton and ion beam therapy. Health policy makers and research funding bodies should also study this overall neutron experience, because it contains many important lessons.

## Conclusions

The following conclusions can be made:

1. It is important that the same fundamental errors that were made with neutrons are not repeated with charged particles. All, rather than some, of the scientific information needs to be included when deciding what may be best to apply in the clinic.
2. As with neutrons, so also with all charged particles, an elevated RBE can be favourable only if the tumour RBE exceeds that of the prescription RBE, but can be a disadvantage when the critical normal tissue RBE exceeds the prescription RBE if accompanied by insufficient normal tissue dose sparing.
3. For any form of radiation therapy, radiobiological testing should be sufficiently comprehensive to identify strengths and weaknesses and include testing in many cell lines and tissues with different radiobiological characteristics and not just a few or one.
4. In retrospect, better radiobiological modelling would have alerted clinicians of at least some of the adverse features and might have prevented the widespread use of fast neutrons, or at least restricted their use to specially defined situations.
5. All new forms of radiotherapy need to be tested in high-quality clinical centres, using the best input from physics, biology, and medicine and use randomised controlled studies wherever possible. These must include rigorous external dosimetry QA and data monitoring committees.
6. The experimental fast neutron database remains important and has implications for proton therapy because neutrons mainly ionise by forming recoil protons.
7. The neutron experimental and clinical experience should be taught to doctors and physicists embarking on careers in radiotherapy and should be studied in greater detail by those engaged in particle beam therapies, as well as health policy decision makers.

## Author Contributions

The author confirms being the sole contributor of this work and has approved it for publication.

## Conflict of Interest

The author declares that the research was conducted in the absence of any commercial or financial relationships that could be construed as a potential conflict of interest.
