# Supplementary material for: Clinical Radiobiology of Fast Neutron Therapy: What Was Learnt?
Source: Front Oncol. 2020 Sep 15;10:1537. doi: 10.3389/fonc.2020.01537 (PMC7522468; doi:10.3389/fonc.2020.01537)
Supplement: Supplementary file 1 [file Data_Sheet_1.docx]

**Appendix**

The RBE_Max_ and RBE_Min_ parameters effectively act as multipliers of the low LET α and β radiosensitivity parameters, and also represent the limits of RBE at low and high dose respectively. These two important parameters have the following identities, where the subscripts L and H refer to low (the reference radiation) and high LET radiations (neutrons or other charged particles) respectively:-

$RBE_{\max}=\frac{\alpha_{H}}{\alpha_{L}}$ [A1], so that $\alpha_{L}=\frac{\alpha_{H}}{R_{\max}}$ [A2]

and $RBE_{\min}^{2}=\frac{\beta_{H}}{\beta_{L}}$ [A3], so that $\beta_{L}=\frac{\beta_{H}}{RBE_{\min}^{2}}$ [A4]

Equation 2 is divided by equation 4, then

$\frac{\alpha_{L}}{\beta_{L}}=\frac{\alpha_{H}}{\beta_{H}}.\frac{RBE_{\min}^{2}}{RBE_{\max}}$ [A5]

Rearrangements of this last equation allow RBE_max_ and RBE_min_ to be expressed as:

$RBE_{\max}=\frac{\alpha_{H}}{\beta_{H}}.\frac{RBE_{\min}^{2}}{\left(\frac{\alpha_{L}}{\beta_{L}}\right)}=\frac{Q}{\left(\frac{\alpha_{L}}{\beta_{L}}\right)}$ [A6]

And

 [A7]

Where $Q=\frac{\alpha_{H}}{\beta_{H}}..RBE_{\min}^{2}$ [A8], and [A9]

An iso-effect obtained separately by a low LET and high LET radiation is provided by the following equation

${d_{L}\left( 1+\frac{d_{L}}{\left( \frac{\alpha}{\beta} \right)_{L}} \right)=d}_{H}\left( RBEmax+\frac{{RBEmin}^{2}d_{H}}{\left( \frac{\alpha}{\beta} \right)_{L}} \right)$ (A10)

The value of *d_H_* is then obtained from the positive root of the above equation which, with *k* replacing (α/β)_L_ as:

$d_{H}=\frac{-k.RBE\max+\sqrt{k^{2}{.RBEmax}^{2}+4{d_{L}}^{2}\mathrm{RBEmin}^{2}+4d_{L}.k.\mathrm{RBEmin}^{2}}}{2RBE\min^{2}}$ (eq13)

The required RBE is then the ratio *d_L_/d_H_*.

The above equations allow estimates of RBE at any dose per fraction and in different tissues and tumours by use of the appropriate low LET α/β ratios. To determine the change in radiosensitivities for specific values of LET, further LET-RBE models are required to provide either a change in α and β or α/β with increasing LET, and so obtain RBEmax and RBEmin as defined in equations A1 and A3 above [38, 39, 45].
